# The Geography of Happiness: Connecting Twitter Sentiment and Expression, Demographics, and Objective Characteristics of Place

**DOI:** 10.1371/journal.pone.0064417

**Published:** 2013-05-29

**Authors:** Lewis Mitchell, Morgan R. Frank, Kameron Decker Harris, Peter Sheridan Dodds, Christopher M. Danforth

**Affiliations:** 1 Computational Story Lab, Department of Mathematics and Statistics, Vermont Complex Systems Center, and the Vermont Advanced Computing Core, The University of Vermont, Burlington, Vermont, United States of America; 2 Department of Applied Mathematics, University of Washington, Seattle, Washington, United States of America; Universidad Carlos III de Madrid, Spain

## Abstract

We conduct a detailed investigation of correlations between real-time expressions of individuals made across the United States and a wide range of emotional, geographic, demographic, and health characteristics. We do so by combining (1) a massive, geo-tagged data set comprising over 80 million words generated in 2011 on the social network service Twitter and (2) annually-surveyed characteristics of all 50 states and close to 400 urban populations. Among many results, we generate taxonomies of states and cities based on their similarities in word use; estimate the happiness levels of states and cities; correlate highly-resolved demographic characteristics with happiness levels; and connect word choice and message length with urban characteristics such as education levels and obesity rates. Our results show how social media may potentially be used to estimate real-time levels and changes in population-scale measures such as obesity rates.

## Introduction

With vast quantities of real-time, fine-grained data, describing everything from transportation dynamics and resource usage to social interactions, the science of cities has entered the realm of the data-rich fields. While much work and development lies ahead, opportunities for quantitative study of urban phenomena are now far more broadly available to researchers [1]. With over half the world’s population now living in urban areas, and this proportion continuing to grow, cities will only become increasingly central to human society [2]. Our focus here concerns one of the many important questions we are led to continuously address about cities: how does living in urban areas relate to well-being? Such an undertaking is part of a general program seeking to quantify and explain the evolving cultural character–the stories–of cities, as well as geographic places of larger and smaller scales.

Numerous studies on well-being are published every year. The UN’s 2012 World Happiness Report attempts to quantify happiness on a global scale with a ‘Gross National Happiness’ index which uses data on rural-urban residence and other factors [3]. In the US, Gallup and Healthways produce a yearly report on the well-being of different cities, states and congressional districts [4], and they maintain a well-being index based on continual polling and survey data [5]. Other countries are also beginning to produce measures of well-being: in 2012, surveys measuring national well-being and how it relates to both health and where people live were conducted in both the United Kingdom by the Office of National Statistics [6], [7] and in Australia by Fairfax Media and Lateral Economics [8].

While these and other approaches to quantifying the sentiment of a city as a whole rely almost exclusively on survey data, there are now a range of complementary, remote-sensing methods available to researchers. The explosion in the amount and availability of data relating to social media in the past 10 years has driven a rapid increase in the application of data-driven techniques to the social sciences and sentiment analysis of large-scale populations.

Our overall aim in this paper is to investigate how geographic place correlates with and potentially influences societal levels of happiness. In particular, after first examining happiness dynamics at the level of states, we will explore urban areas in the United States in depth, and ask if it is possible to (a) measure the overall average happiness of people located in cities, and (b) explain the variation in happiness across different cities. Our methodology for answering the first question uses word frequency distributions collected from a large corpus of geolocated messages or ‘tweets’ posted on Twitter, with individual words scored for their happiness independently by users of Amazon’s Mechanical Turk service [9]. This technique was introduced by Dodds and Danforth (2009) [10] and greatly expanded upon in Dodds et al. (2011) [11], as well as tested for robustness and sensitivity. In attempting to answer the second question of happiness variability, we examine how individual word usage correlates with happiness and various social and economic factors. To do this we use the ‘word shift graph’ technique developed in [10], [11], as well as correlate word usage frequencies with traditional city-level census survey data. As we will show, the combination of these techniques produces significant insights into the character of different cities and places.

We structure our paper as follows. In the Methods section, we describe the data sets and our methodology for measuring happiness. In part 1 of the Results section we measure the happiness of different states and cities and determine the happiest and saddest states and cities in the US, with some analysis of why places vary with respect to this measure. In part 2 of the Results section we compare our results for cities with census data, correlating happiness and word usage with common social and economic measures. We also use the word frequency distributions to group cities by their similarities in observed word use. We conclude with a discussion of the results and outlook for further research.

## Methods

We examine a corpus of over 10 million geotagged tweets gathered from 373 urban areas in the contiguous United States during the calendar year 2011. This corpus is a subset of Twitter’s ‘garden hose’ feed, which in 2011 represented roughly 10% of all messages. For the present study, we focus on the approximately 1% of tweets that are geotagged. Urban areas are defined by the 2010 United States Census Bureau’s MAF/TIGER (Master Address File/Topologically Integrated Geographic Encoding and Referencing) database [12]. Note that these urban area boundaries often agglomerate small towns together, particularly when there are small towns geographically close to larger towns or cities. See Appendix A in Appendix S1 for a more detailed description of the data set as well as an exploration of the relationship between area and perimeter, or fractal dimension, of these cities.

To measure sentiment (hereafter happiness) in these areas from the corpus of words collected, we use the Language Assessment by Mechanical Turk (LabMT) word list (available online in the supplementary material of [11]), assembled by combining the 5,000 most frequently occurring words in each of four text sources: Google Books (English), music lyrics, the New York Times and Twitter. A total of roughly 10,000 of these individual words have been scored by users of Amazon’s Mechanical Turk service on a scale of 1 (sad) to 9 (happy), resulting in a measure of average happiness for each given word [13]. For example, ‘rainbow’ is one of the happiest words in the list with a score of 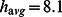, while ‘earthquake’ is one of the saddest, with 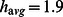. Neutral words like ‘the’ or ‘thereof’ tend to score in the middle of the scale, with 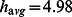 and 5 respectively.

For a given text *T* containing *N* unique words, we calculate the average happiness 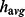 by
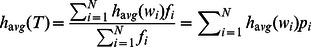
(1)where 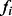 is the frequency of the *i*th word 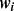 in *T* for which we have a happiness value 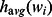, and 
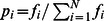
 is the normalized frequency of word 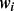.

Importantly, with this method we make no attempt to take the context of words or the meaning of a text into account. While this may lead to difficulties in accurately determining the emotional content of small texts, we find that for sufficiently large texts this approach nonetheless gives reliable (if eventually improvable) results. An analogy is that of temperature: while the motion of a small number of particles cannot be expected to accurately characterize the temperature of a room, an average over a sufficiently large collection of such particles nonetheless defines a durable quantity. Furthermore, by ignoring the context of words we gain both a computational advantage and a degree of impartiality; we do not need to decide *a priori* whether a given word has emotional content, thereby reducing the number of steps in the algorithm and hopefully reducing experimental bias.

Following Dodds et al. (2011), for the remainder of this paper, we remove all words 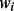 for which the happiness score falls in the range 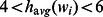 when calculating 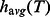. Removal of these neutral or ‘stop’ words has been demonstrated to provide a suitable balance between sensitivity and robustness in our ‘hedonometer’ [11]. Further details on how we preprocessed the Twitter data set can be found in Appendix A in Appendix S1.

We will correlate our happiness results with census data which was taken from the 2011 American Community Survey 1-year estimates, accessible online at http://factfinder2.census.gov/.

## Results

### 1 Happiness across States and Urban Areas

**Figure 1:**
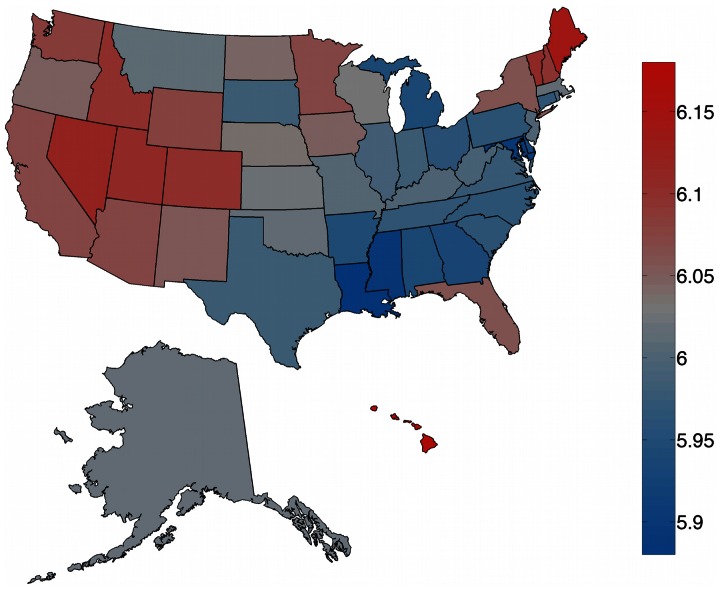
Average word happiness for geotagged tweets in all US states collected during calendar year 2011. The happiest 5 states, in order, are: Hawaii, Maine, Nevada, Utah and Vermont. The saddest 5 states, in order, are: Louisiana, Mississippi, Maryland, Delaware and Georgia. Word shift plots describing how differences in word usage contribute to variation in happiness between states are presented in Appendix B in Appendix S1 (online) [19].

We first examine how happiness varies on a somewhat coarser scale than we will focus on for the majority of this paper, by plotting the average happiness of all states in the US in Figure 1. To avoid the problem that some states have happier names than others, we removed each state name from the calculation for 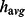. We also removed instances of the capitalized string ‘HI’, which generally occurred as the state code for Hawaii and positively biased the score for that state. We remark however that including this string increased Hawaii’s score by only 0.2%; in general we find that the hedonometer is very robust to small variations in word frequencies such as this.

At such a coarse resolution there is little variation between states, which all lie between 0.15 of the mean value for the entire United States of 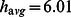. The happiest state is Hawaii with a score of 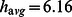 and the saddest state is Louisiana with a score of 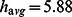. The complete list for all states can be found in Table S1 in Appendix S1. Hawaii emerges as the happiest state due to an abundance of relatively happy words such as ‘beach’ and food-related terms. A similar result showing greater happiness and a relative abundance of food-related words in tweets made by users who regularly travel large distances (as would be the case for many of the tweets emanating from Hawaii) has been reported in [3]. Louisiana is revealed as the saddest state, with a significant factor being an abundance of profanity relative to the other states. This is in contrast with the findings of Oswald and Wu [15], [16], who determined Louisiana to be the state with highest well-being according to an alternate survey-based measure of life satisfaction.

**Figure 2:**
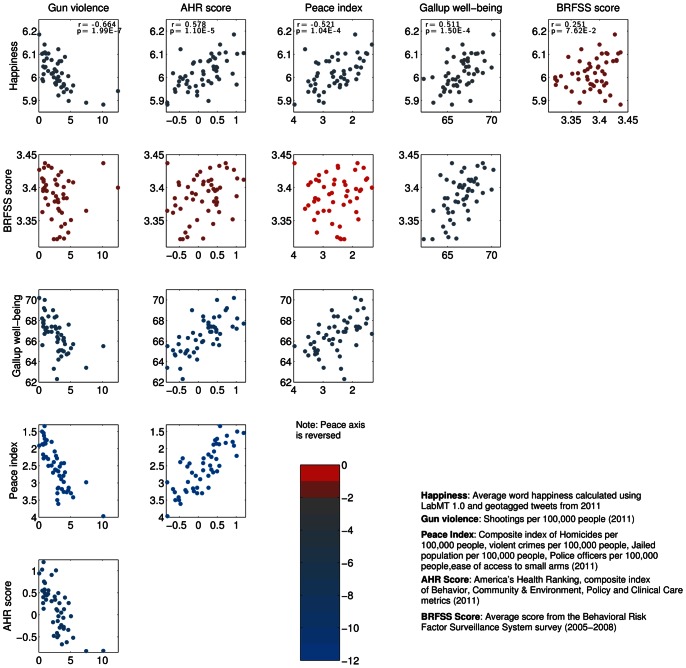
Scatter plot matrix of correlations between different well-being measures. Points are colored by *p*-value, statistically insignificant correlations above 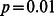 are shown in red. Spearman’s *r* and *p*-value are reported in the inset.

In Figure 2 we compare our results with five other well-being measures:

- the behavioral risk factor survey score (BRFSS) used by Oswald and Wu [16], a survey of life satisfaction across the United States;
- the 2011 Gallup well-being index [17], based on survey data about life evaluation, emotional and physical health, healthy behavior, work environment and basic access;
- the 2011 United States peace index [17] produced by the Institute for Economics and Peace, a composite index of homicides per 100,000 people, violent crimes per 100,000 people, size of jailed population per 100,000 people, number of police officers per 100,000 people, and ease of access to small arms;
- the 2011 United Health Foundation’s America’s health ranking (AHR) [18], a composite index of behavior, community and environment, policy, and clinical care metrics;
- the number of shootings per 100,000 people in 2011.

Figure 2 shows a matrix of scatter plots labelled with the correlations between each of the above measures, including average word happiness. Spearman’s correlation coefficient *r* and *p*-values are reported in the inset for each scatter plot. Points are colored by *p*-value, with blue points indicating stronger correlation and red indicating insignificant correlations above 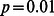. Our measure of state happiness (top row) correlates strongly with all other measures except for the BRFSS, however the BRFSS itself correlates significantly only with the Gallup well-being index. Possible explanations for the poor agreement between BRFSS and the other measures may include its placing of Louisiana at the top of the well-being list, which is generally opposite to its position in similar lists. The BRFSS also uses data collected between 2005 and 2008, whereas all the other lists use data from 2011 only.

**Figure 3:**
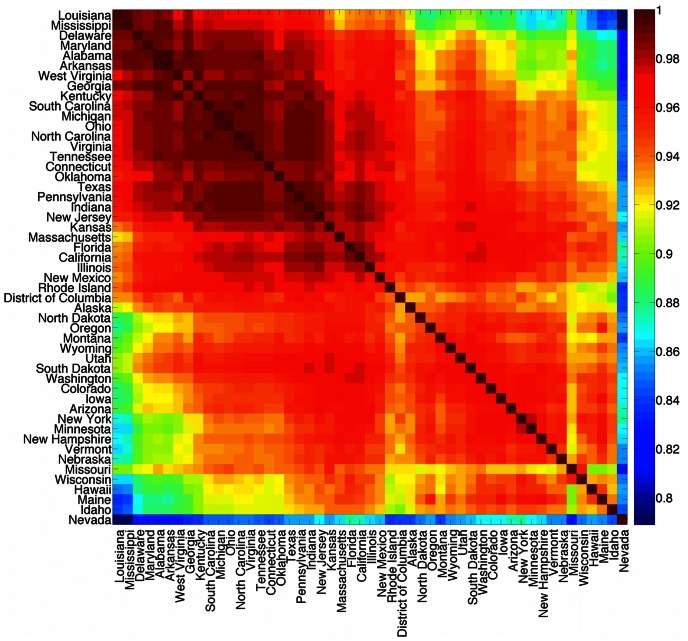
Clustergram showing cross-correlations between word frequency distributions for all states in 2011. Red signifies states with similar or highly-correlating word frequency distributions, while blue signifies states with relatively dissimilar word frequency distributions.

We can further use this data on word frequencies to characterize similarities between states based on word usage. For simplicity, we focus on the 50,000 most frequently occurring words on Twitter [11]. Figure 3 shows the linear correlation between word frequency vectors 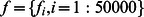 for each pair of states, with red entries in the matrix indicating states with similar word use. We see some clusters which might be explained by geographical proximity, such as Vermont and New Hampshire or Louisiana and Mississippi, and some outliers such as the state of Nevada, which correlates the lowest on average with all other states. Additional details on this state-level dataset, including plots of raw number of tweets and number of tweets per head of population for each state can be found in Appendix A in Appendix S1. Word shift graphs showing which words contribute most to the variation in happiness across states can be found in Appendix B in Appendix S1 (online) [19].

**Figure 4:**
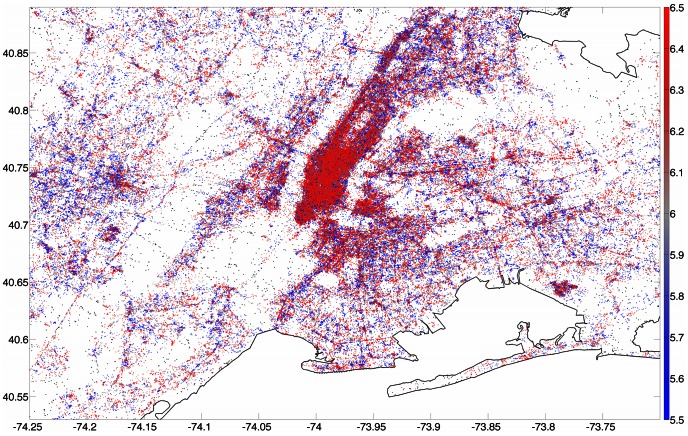
Map of tweets collected from New York City during the calendar year 2011. Each point represents an individual tweet and is colored by the average word happiness 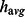 of nearby tweets: red is happier, blue is sadder. For a point to be colored, we require that there be at least 200 LabMT words within a 500 meter radius of the location; points which do not satisfy this criterion are colored black. Maps for all other cities can be found in Appendix C in Appendix S1 (online) [19].

We now change our resolution to a finer scale by focussing on cities rather than states. As an illustration of the resolution of the data set as well as our technique, we plot a tweet-generated map of a city, showing how average word happiness varies with location. In Figure 4 we plot tweets collected from the New York City area during 2011. Each point represents an individual tweet, and is colored by the happiness 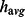 of the text *T* consisting of the 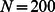 LabMT words contained in the geotagged tweets closest to that location. We set a maximum threshold radius of 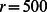 meters within which to find other geotagged tweets around each point; if 200 LabMT words cannot be found within that radius then the point is colored black.

Several features can immediately be discerned in this purely tweet-generated map. Firstly, the spatial resolution reveals the outline of Manhattan, as well as Central Park, individual streets and bridges, and even airport terminals such as those at JFK and Newark airports at the lower right and center left of the figure respectively. Secondly, we can discern regions of higher and lower happiness: the Harlem and Washington Heights areas to the north appear relatively sad compared to the Downtown/Midtown area, as does the Waterfront, New Jersey area west of the southern tip of Manhattan. Similar tweet-generated maps for all 373 cities measured are presented in Appendix B in Appendix S1 (online) [19].

**Figure 5:**
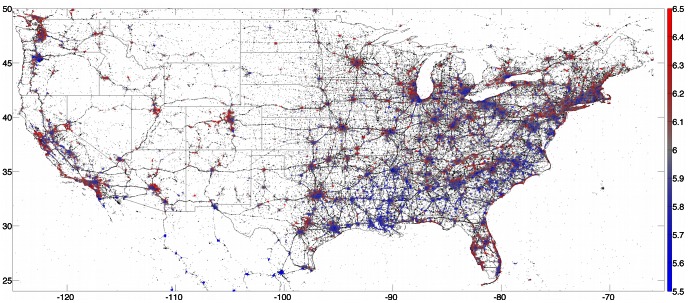
Map showing happiness of all tweets collected from the lower 48 US states during 2011. Points are colored as in figure 4, except we now require that there are at least 500 LabMT words within a 10 kilometer radius of the location of each tweet in order to be colored.

In Figure 5 we show a tweet-generated happiness map of the entire contiguous United States, where we have now used 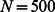 and 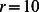 km. We can clearly discern cities and the roads between them at this scale, and substantial variation in happiness across geographical regions. There is already an indication that some cities will be significantly less happy than others, particularly those in the southeastern United States, a conclusion which will be made more quantitative later. At a finer scale we can see that some coastal areas, particularly around the Florida peninsula and along the coast of North and South Carolina, are significantly happier than the regions immediately inland of them. We will see this again below in the word shifts for various oceanside cities. Finally, we remark upon one limitation of the present methodology by noting that the Mexican cities shown in Figure 5 appear far sadder than their counterparts to the north. This is due to the presence of Spanish words such as ‘con’ and ‘sin’, which while neutral in Spanish have been scored as negative English words in LabMT. At present the LabMT list is applicable only to English-language texts; future versions of the list will incorporate scores for languages other than English as well.

**Figure 6:**
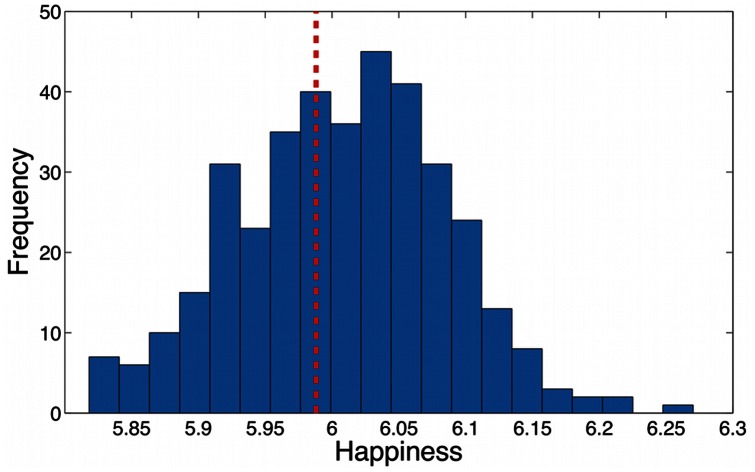
Distribution of average happiness values for all 373 cities in the census data set. A vertical dashed line denotes the average for all cities. Note the greater weight towards the right of the distribution, with more cities having happiness scores higher than the average.

Next we calculate the happiness 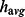 for each city in the census data set using equation (1), where the boundaries of a city are defined by the MAF/TIGER database, and each text *T* is formed by agglomerating all the words falling within that city. Figure 6 shows the distribution of happiness scores for all cities; as is to be expected for smaller samples, the range of values is slightly higher than that calculated for the states, extending over a range of more than 0.2 from the mean of 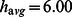. We remark that the distribution is skewed: there are more cities that are happier than the overall average, by 220 to 153.

**Figure 7:**
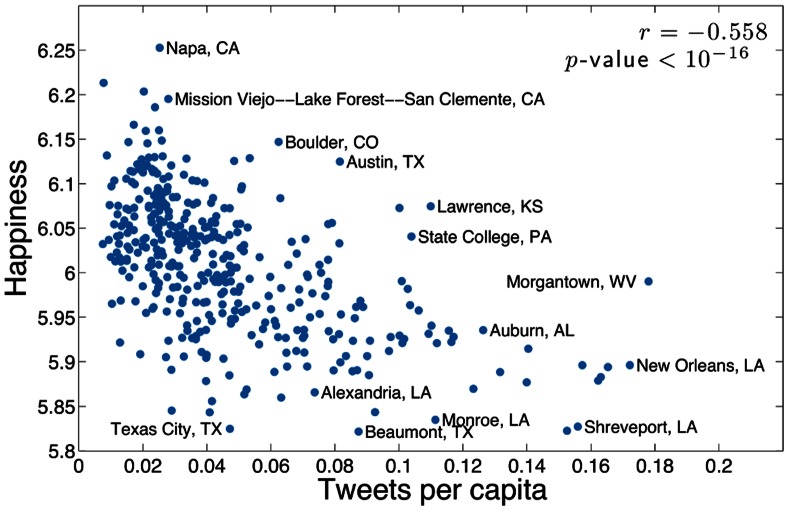
Happiness as a function of number of tweets per capita. Areas with a higher density of tweets per capita tend to be less happy.

It is well known that city population sizes follow a power law distribution (see [20] and many others), which in conjunction with Figure 6 suggests that happiness decreases with city size. While we do find a slight negative correlation between happiness and the number of tweets gathered in each city, we in fact find that happiness more strongly negatively correlates with the number of tweets per capita, with Spearman correlation coefficient −0.558 and *p*-value less than 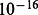, as shown in Figure 7.

**Figure 8:**
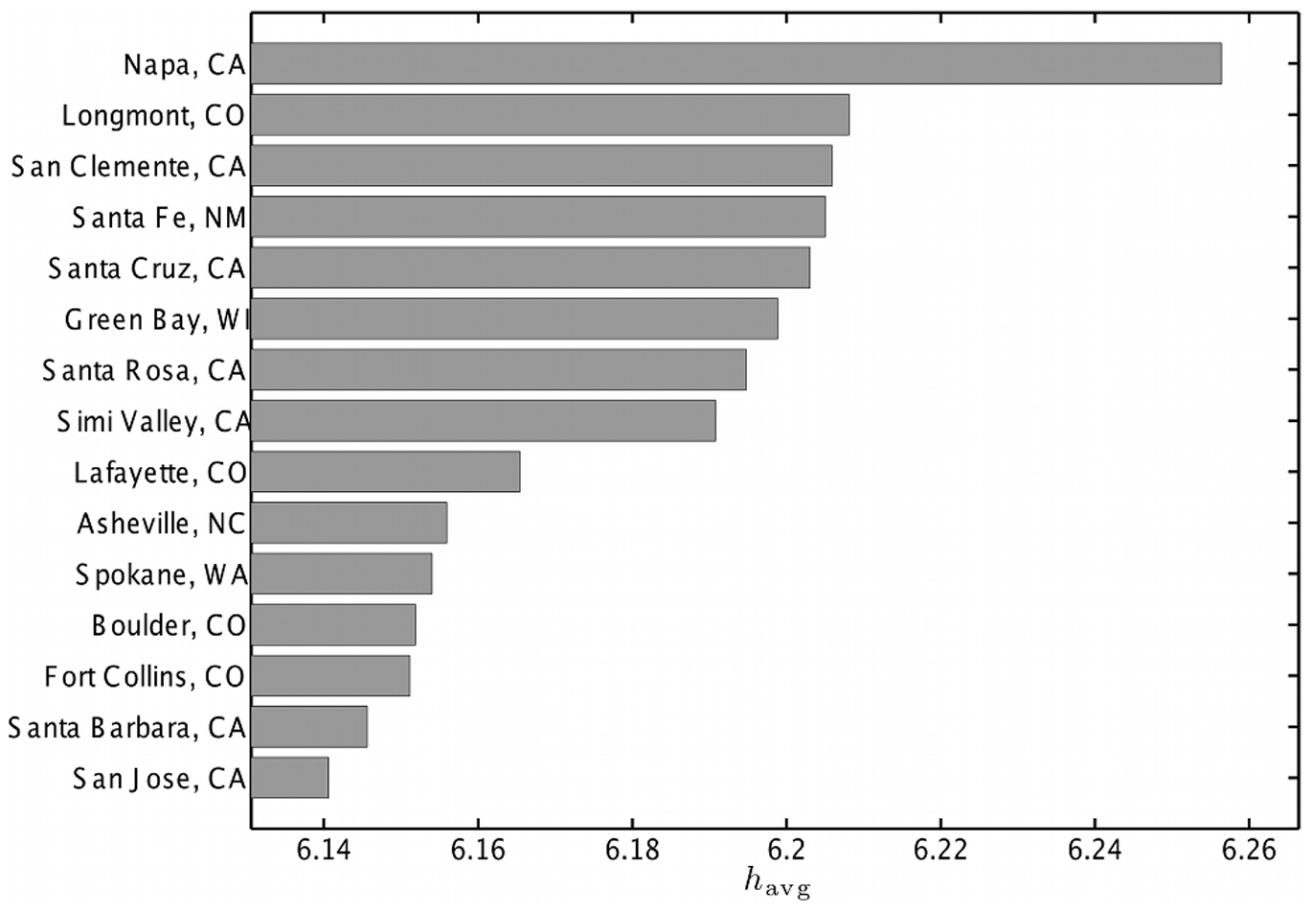
The 15 highest average word happiness scores 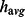 for cities in the contiguous USA. Scores were calculated using (1) and the LabMT word list. The full list of cities can be found in Appendix C in Appendix S1 (online) [19].

**Figure 9:**
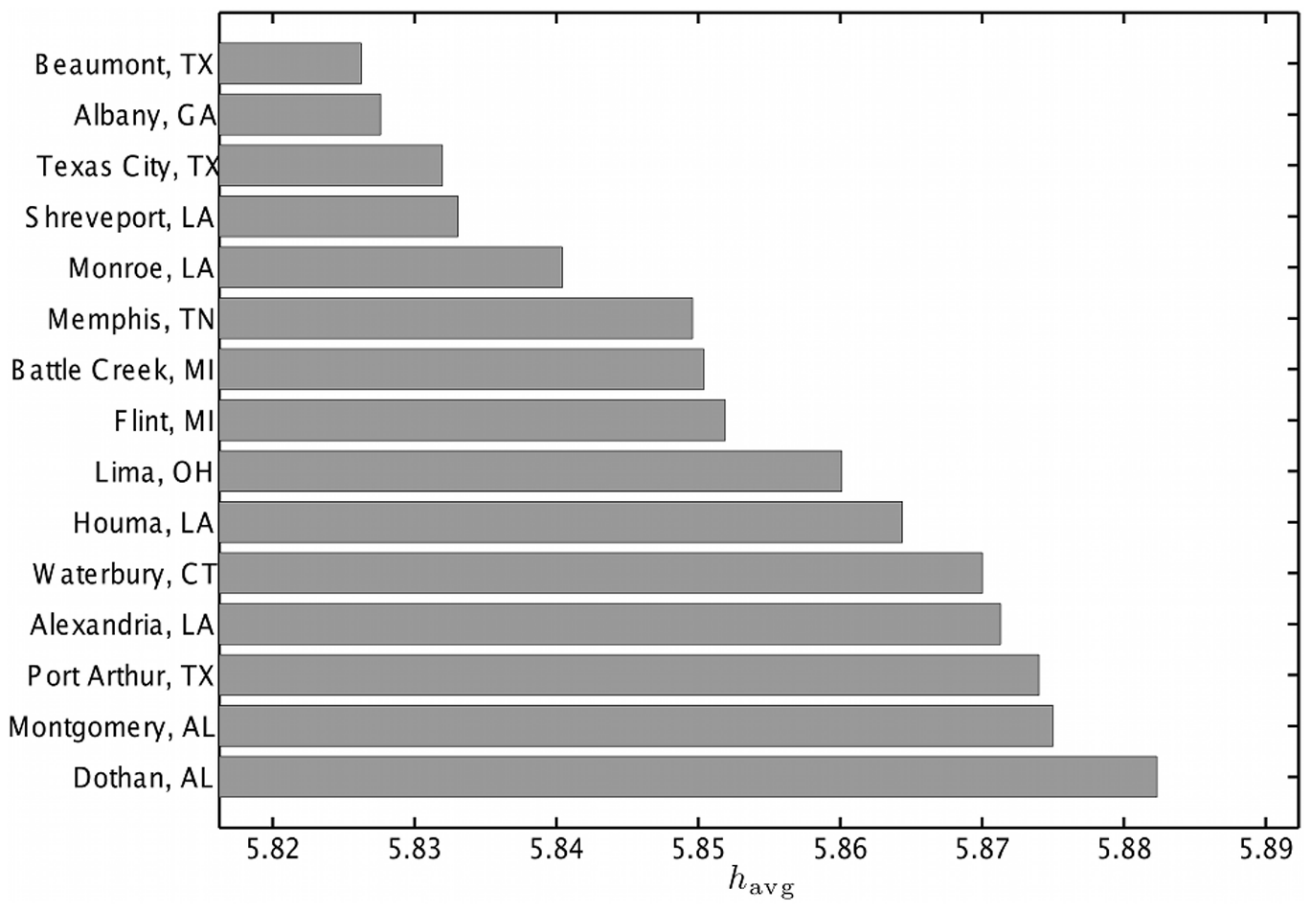
The 15 lowest average word happiness scores 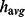 for cities in the contiguous USA. Scores were calculated using (1) and the LabMT word list. The full list of cities can be found in Appendix C in Appendix S1 (online) [19].

The bar charts in Figures 8 and 9 show the average word happiness 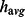 for the 15 happiest and 15 saddest cities in the contiguous United States, respectively. Using this method we identify Napa, California as the happiest city in the US with a score of 6.26, and Beaumont, Texas as the saddest city with a score of 5.83.

As was the case with our state happiness rankings, several cities that ranked both highly and lowly by our measure rank similarly in more traditional survey based efforts. For example, the 2011 Gallup-Healthways well-being survey [4] showed Boulder, Colorado as the city with the fifth highest well-being index composite score (and twelfth highest happiness score in our list), while Flint, Michigan had the second lowest and Montgomery, Alabama the 21st-lowest well-being index (compared to 8th lowest and 14th lowest happiness scores on our list). The overall Spearman correlation between the rankings using Gallup’s well-being index and our measure is 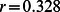, with *p*-value 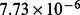 (a scatter plot is presented online in Appendix C in Appendix S1). Whereas our list uses only word frequencies in the calculation of 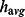, the Gallup-Healthways score is an average of six indices which measure life evaluation, emotional health, work environment, physical health, healthy behaviors, and access to basic necessities. We remark that our method is far more efficient to implement than a survey-based approach, and it provides a near real-time stream of information quantifying well-being in cities.

**Figure 10:**
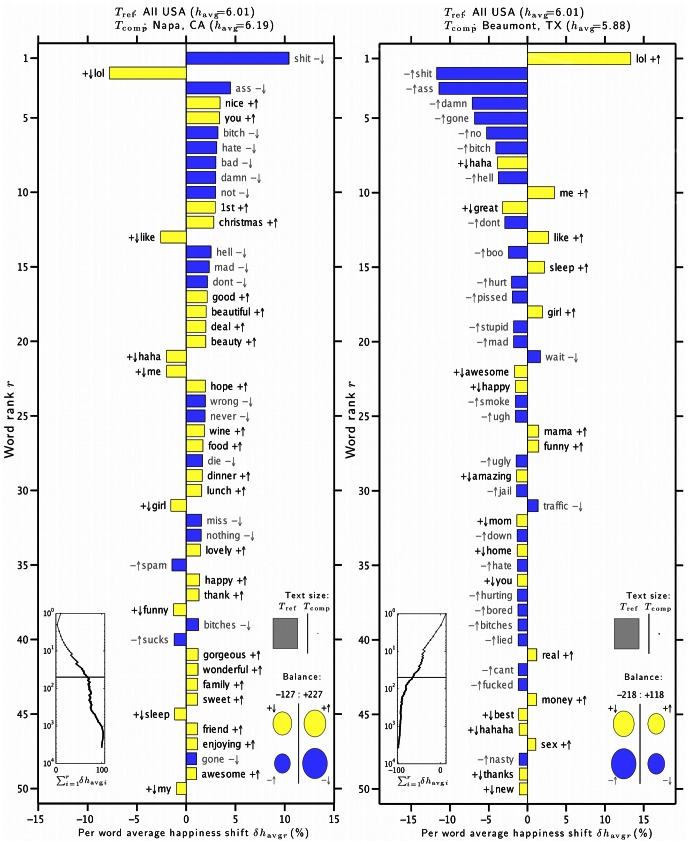
Word shift graphs for the happiest city and saddest city. These show how 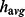 varies for all US cities considered versus the cities Napa, California (left) and Beaumont, Texas (right), having the highest and lowest 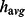 respectively. Words are ranked in order of decreasing percentage contribution to the overall average happiness difference 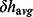. The symbols 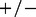 indicate whether a word is relatively happy or sad compared to 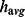 for the entire US (text 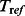), while the arrows 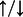 indicate whether the word was used more or less in the text 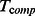 for each city than in 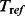. The left inset panel shows how the ranked LabMT words combine in sum. The four circles at bottom right show the total contribution of the four kinds of words (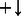, 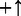, 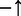, 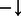). Relative text size is indicated by the areas of the gray squares.

To investigate why the average word happiness varies across urban areas, we study the word shift graphs [10], [11] for each city. These graphs show how the difference in happiness for two texts depends on differences in the underlying word frequencies. In Figure 10 we show the word shift graphs for Napa and Beaumont, as compared to the entire corpus of words collected for all urban areas during 2011. Word shift graphs for every city are presented in Appendix C in Appendix S1 (online) [19].

We observe some features of the graphs that are consistent with geography–for example the word ‘beach’ appears high on the list of words for coastal cities such as Santa Cruz, California or Miami, Florida. Overall, the main factor driving the relative happiness scores for each city appears to be the presence or absence of key words such as ‘lol’, ‘haha’ and its variants, ‘hell’, ‘love’, ‘like’ and the negative words ‘no’, ‘don’t’, ‘never’ and ‘wrong’, as well as profanity.

### 2 Correlating Word Usage with Census Data

The word shifts of Figure 10 demonstrate how word usage varies with location, as well as the importance of studying the individual words that go in to the calculation of averaged quantities such as the word happiness 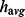. We therefore now examine in greater detail how happiness and word usage relate to underlying social factors.

We first focus on how the average happiness 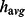 correlates with different social and economic measures. To do this we took data from the 2011 American Community Survey 1-year estimates, specifically tables DP02 through DP05 covering selected social characteristics, economic characteristics, housing characteristics and demographic and housing estimates. These tables contained 508 different categories for all cities, from which we removed the categories with data on less than 75% of all cities, leaving 432 different categories for correlation with happiness.

**Figure 11:**
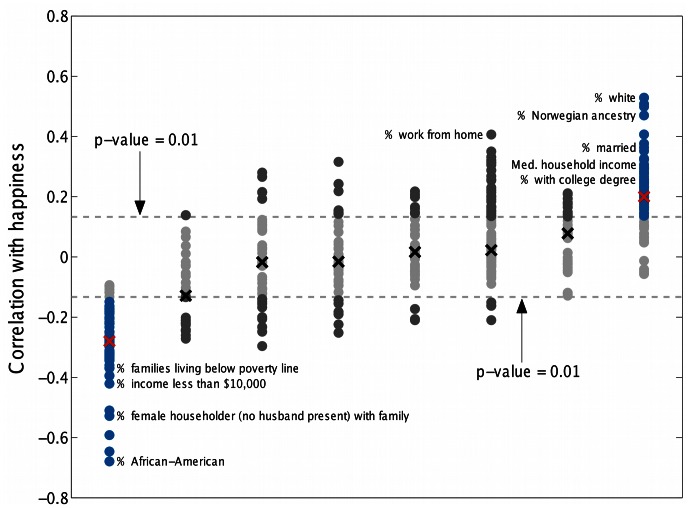
Spearman correlations for 432 demographic attributes with happiness. The 8 groupings along the horizontal axis are for covarying attributes identified by agglomerative hierarchical clustering, independently of happiness. Crosses lie on the median of each cluster, and the dashed lines represent the 1% significance level. The two clusters which have medians that correlate significantly with happiness are colored blue. A complete list of the correlation of all attributes with happiness can be found in Appendix D in Appendix S1 (online) [19].

In Figure 11 we show the Spearman correlation between happiness and each demographic attribute for all 373 cities. Each point in the graph represents one of the 432 attributes considered; a table listing each demographic and its correlation with happiness is presented in Appendix D in Appendix S1 (online) [19]. The groupings into columns were made independently of happiness values, by performing complete-link clustering using a hierarchical cluster tree on the table of census attributes for all cities [21]. The 8 clusters found are not unique and depend on the distance threshold used, however they give some indication of which attributes covary. Only two groups show a large number of attributes which significantly correlate (below 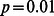) with happiness; these are shown in blue (with red crosses specifying the median attribute). These two groups might be broadly characterized as representing high socioeconomic and low socioeconomic status respectively, with many of the attributes in the high socioeconomic status group positively correlating with happiness, and anti-correlating for the low socioeconomic status group.

**Figure 12:**
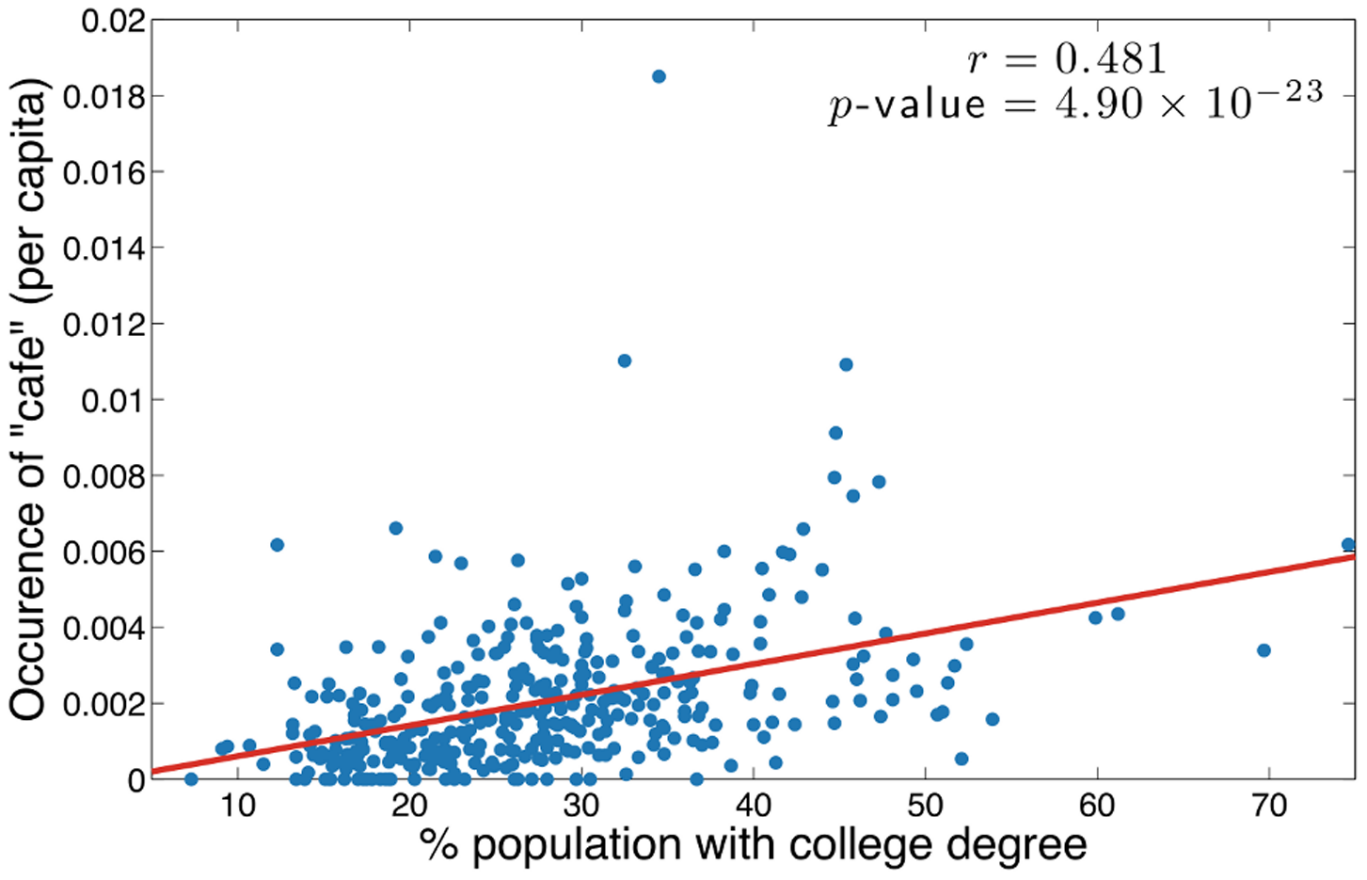
Correlation between education and use of the word ‘café’. The scatter plot shows the correlation between rate of occurrence of the word ‘café’ and percentage of population with a bachelor’s degree or higher in US cities during the calendar year 2011. The red line shows linear correlation while the reported *r* and *p*-values show the Spearman correlation.

To further understand what drives this correlation of certain demographics with happiness, we now investigate how each word from the LabMT list correlates with each census attribute. To do this we first normalize the word counts in each urban area by the total number of tweets collected in each city, and then for each word calculate the Spearman correlation *r* between normalized frequency and census attribute for all cities. For example, the scatter plot in Figure 12 shows that the normalized frequency of occurrence of the word ‘café’ shows a strong positive correlation with the percentage of the population with a bachelors degree or higher. The Spearman correlation between the two is 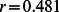 with *p*-value 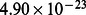, indicating strong correlation.

We present lists showing the correlation of each LabMT word with every demographic attribute in Appendix D in Appendix S1 (online) [19]. Taking the percentage of population with a bachelors degree or higher as a representative example, Tables 1 and 2 show the top 25 words which exhibit the highest positive and negative correlations respectively with this attribute. We note that the positive correlations in Table 1 are much stronger than the negative correlations in Table 2; a similar asymmetry appears in many of the tables in Appendix D in Appendix S1. The results show that longer words such as ‘software’, ‘development’ and ‘emails’ correlate strongly with high levels of education, while the words which correlate negatively with education are generally shorter, with no words longer than two syllables appearing in the list. Furthermore, many of the words such as ‘love’, ‘talk’ and ‘mom’ appearing in Table 2 are family- or relationship-oriented, while the words in Table 1 are generally more employment-oriented, and suggest more complex and abstract intellectual themes. It may be postulated that this is a reflection of the social processes occurring in urban areas characterized by low and high education rates, respectively.

**Table 1:** Words showing strongest positive correlation with education.

Word	*r*	*p*-value	*h_avg_* (*w_i_*)
cafe	0.481	4.9×10^−23^	6.78
pub	0.463	3.14×10^−21^	6.02
software	0.458	9.07×10^−21^	6.30
yoga	0.455	1.85×10^−20^	7.04
grill	0.433	1.78×10^−18^	6.24
development	0.424	1.14×10^−17^	6.38
emails	0.419	2.87×10^−17^	6.54
wine	0.417	3.83×10^−17^	6.42
library	0.414	6.47×10^−17^	6.48
art	0.414	6.8×10^−17^	6.60
sciences	0.410	1.54×10^−16^	6.30
pasta	0.410	1.57×10^−16^	6.86
lounge	0.409	1.68×10^−16^	6.50
market	0.408	2.2×10^−16^	6.28
india	0.407	2.5×10^−16^	6.42
drinking	0.405	3.74×10^−16^	6.14
technology	0.405	3.76×10^−16^	6.74
forest	0.405	3.83×10^−16^	6.68
brunch	0.405	3.89×10^−16^	6.32
dining	0.403	4.92×10^−16^	6.48
supporting	0.399	1.1×10^−15^	6.48
professor	0.398	1.23×10^−15^	6.04
university	0.392	3.62×10^−15^	6.74
film	0.391	4.27×10^−15^	6.56
global	0.391	4.72×10^−15^	6.00

Top 25 words with strongest positive Spearman correlation *r* to percentage of population with a Bachelors degree or higher (census table DP02-HC03-VC94) in 2011. Stop words with 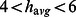 have been removed from the list. Note the low *p*-values for all words, indicating strong statistical significance.

**Table 2:** Words showing strongest negative correlation with education.

Word	*r*	*p*-value	*h_avg_* (*w_i_*)
me	−0.393	3.26×10^−15^	6.58
love	−0.389	6.51×10^−15^	8.42
my	−0.354	1.97×10^−12^	6.16
like	−0.346	6.04×10^−12^	7.22
hate	−0.344	8.76×10^−12^	2.34
tired	−0.343	1×10^−11^	3.34
sleep	−0.341	1.27×10^−11^	7.16
stupid	−0.328	8.55×10^−11^	2.68
bored	−0.315	5.11×10^−10^	3.04
you	−0.315	5.23×10^−10^	6.24
goodnight	−0.305	1.77×10^−9^	6.58
bitch	−0.295	6.51×10^−9^	3.14
all	−0.289	1.33×10^−8^	6.22
lie	−0.285	2.24×10^−8^	2.60
mom	−0.284	2.42×10^−8^	7.64
wish	−0.271	1.05×10^−7^	6.92
talk	−0.267	1.74×10^−7^	6.06
she	−0.265	2.01×10^−7^	6.18
know	−0.262	2.78×10^−7^	6.10
ill	−0.259	4.11×10^−7^	2.42
dont	−0.258	4.54×10^−7^	3.70
well	−0.256	5.3×10^−7^	6.68
don’t	−0.255	5.8×10^−7^	3.70
give	−0.255	5.84×10^−7^	6.54
friend	−0.255	6.27×10^−7^	7.66

Top 25 words with strongest negative Spearman correlation *r* to percentage of population with a Bachelors degree or higher in 2011 (with stop words removed).

The technique applied here is not limited only to census data. As an example of a different use of the corpus, we now correlate word use to obesity at the metropolitan level. For this study we take obesity levels from the Gallup and Healthways 2011 survey [22], and metropolitan areas as defined by the U.S. Office of Management and Budget’s Metropolitan Statistical Areas (MSAs) [23]. These MSAs are generally two to three times larger in area than the TIGER urban area census boundaries, and the Gallup obesity survey was only for the 190 largest-population areas. The obesity data set therefore contains fewer small cities than the TIGER census set does, particularly in the Midwest. We collected more than 10 million tweets from these 190 MSAs, corresponding to just over 80 million words during 2011.

**Figure 13:**
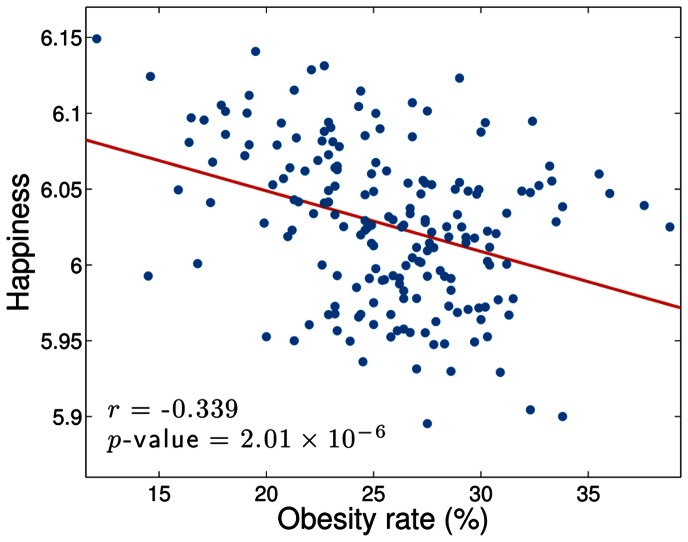
Correlation between happiness and obesity. The scatter plot shows the correlation between 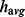 and obesity level, as taken from the 2011 Gallup and Healthways survey. The red line is the straight line of best fit to the data, while the *r* value is the Spearman correlation coefficient for the data.

Performing the same analysis as for the attributes in Figure 11, in Figure 13 we show the relationship between happiness and obesity for the 190 MSAs included in the Gallup survey. We find that happiness generally decreases as obesity increases, with the third happiest city in this set (Boulder, Colorado) corresponding with the lowest obesity rate (12.1%) and the saddest city (Beaumont, Texas, as found previously) corresponding with the fifth highest obesity rate (33.8%). We calculate a Spearman correlation coefficient (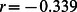 with *p*-value 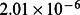) which indicates statistically significant negative correlation between obesity and happiness.

As we did for the census data, we also correlate the abundance of each individual word in the LabMT list to obesity levels in the 190 cities surveyed. From this list we extract words that are clearly food-related, and in Table 3 present those which most most strongly correlate (both negatively and positively) with obesity. Note that we are including stop words for which 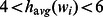 in these lists. Coffee-related words such as ‘café’, ‘coffee’, ‘espresso’ and ‘bean’ feature prominently in the list, and many of the words refer to eating at restaurants–‘sushi’, ‘restaurant’, ‘cuisine’ and ‘brunch’, for example. As we might expect such words to correlate with wealth, this suggests a correlation between obesity and poverty, a claim which we note remains contentious in the medical literature (for example, supported in [24], [25], and refuted in [26]).

**Table 3:** Food-related words showing strongest positive and negative correlations with obesity.

Word	*r*	*p*-value	*H_avg_*(*w_i_*)
cafe	−0.509	6.07×10^−14^	6.78
sushi	−0.487	9.93×10^−13^	5.40
brewery	−0.469	8.67×10^−12^	N/A
restaurant	−0.448	8.93×10^−11^	7.06
bar	−0.435	3.59×10^−10^	5.82
banana	−0.434	3.77×10^−10^	6.86
apple	−0.408	5.22×10^−9^	7.44
fondue	−0.403	8.34×10^−9^	N/A
wine	−0.400	1.08×10^−8^	6.42
delicious	−0.392	2.17×10^−8^	7.92
dinner	−0.386	3.85×10^−8^	7.40
coffee	−0.384	4.51×10^−8^	7.18
bakery	−0.383	5.12×10^−8^	N/A
bean	−0.378	7.88×10^−8^	5.80
espresso	−0.377	8.47×10^−8^	N/A
cuisine	−0.376	8.82×10^−8^	N/A
foods	−0.374	1.07×10^−7^	7.26
tofu	−0.372	1.27×10^−7^	N/A
brunch	−0.368	1.79×10^−7^	6.32
veggie	−0.364	2.46×10^−7^	N/A
organic	−0.361	3.13×10^−7^	6.32
booze	−0.360	3.34×10^−7^	N/A
grill	−0.354	5.4×10^−7^	6.24
chocolate	−0.351	6.77×10^−7^	7.86
#vegan	−0.350	7.47×10^−7^	N/A
mcdonalds	0.246	6.18×10^−4^	5.98
eat	0.241	8.22×10^−4^	7.04
wings	0.222	2.13×10^−3^	6.52
hungry	0.210	3.65×10^−3^	3.38
heartburn	0.194	7.37×10^−3^	N/A
ham	0.177	1.45×10^−2^	5.66

The top 25 food-related words only with strongest negative correlation to obesity level (top), and the 6 food-related words with positive correlation to obesity level and *p*-value less than 0.05 (bottom).

Conversely, only 6 food-related words significantly positively correlate with obesity with *p*-values less than 0.05 (note again the asymmetry in the number of words which positively and negatively correlate with obesity). The fast food chain ‘mcdonalds’ correlates most strongly, and the foods ‘wings’ and ‘ham’ both appear. Unlike in the low-obesity word table, words describing a desire for food–‘eat’ and ‘hungry’–as well as the negative reaction of ‘heartburn’ to overeating, both appear on the list. In Appendix A in Appendix S1 we show tables listing the food-related words which show the least correlation with obesity (Tables S2 and S3 in Appendix S1), as well as the top 25 words (food-related or not) from the LabMT list that correlate and anti-correlate with obesity (Table S4 in Appendix S1). The full list of LabMT words and their correlations with obesity can be found in Appendix E in Appendix S1 (online) [19].

The above analysis demonstrates that different cities have unique characteristics. We now ask whether cities can be sorted into groups based solely upon similarities in their word distributions. Bettencourt *et al.* [27] used data on the economy, crime and innovation to characterize cities; here we use a similar methodology except with word frequency data to uncover so-called ‘kindred’ cities.

**Figure 14:**
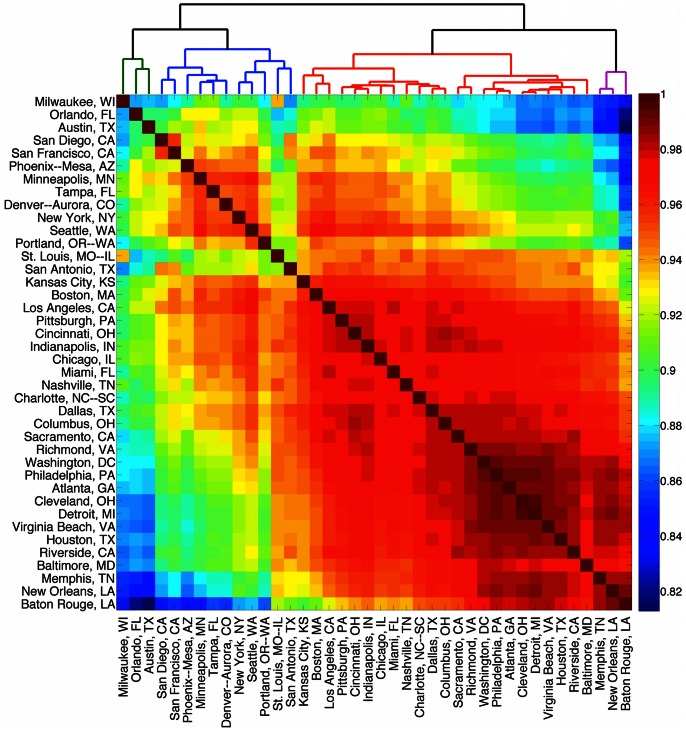
Cross-correlations between word frequency distributions for 40 cities. The clustergram shows Cross-correlations between word frequency distributions for the 40 cities with highest word counts in 2011. Red signifies cities with similar word frequency distribution, while blue signifies cities with dissimilar word frequency distributions.

We group the top 40 cities with highest total word counts in 2011 by calculating the linear correlation between word frequency vectors **f** as we did in Figure 3. The resulting cross-correlation matrix is shown in Figure 14, with red signifying strong correlation between cities. Firstly we note that all cities show similar word frequency distributions, with all correlations being higher than 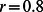. As was the case for the states (see Figure 3), we see one clear large group of strongly correlated cities emerge in the lower right corner, with a smaller distinct cluster appearing at the top left. Perhaps uniquely, these groupings are defined solely by similarities in word usage between cities, rather than by geography or economic indicators.

We cluster cities using an agglomerative hierarchical method with average linkage clustering [21], as shown in the dendrogram at the top of Figure 14, and highlight the 4 clusters with lowest linkage threshold using different colors. As one might expect, some cities that are geographically nearby are grouped together. Notable examples are the Southern cities of Baton Rouge, New Orleans and Memphis in the lower right of the plot, as well as the Californian cities of San Diego and San Francisco at top left. However, this pattern does not hold for all cities; while there is the suggestion of a north/south grouping between the two clusters at the top left and the two at the bottom right, some cities such as Austin and Tampa in the south and Detroit and Philadelphia in the north go against this trend. The cities of Cleveland and Detroit are the most alike in word use, having a cross-correlation of 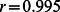, while Austin and Baton Rouge are the most dissimilar with a cross-correlation of 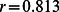. Indianapolis is the city with highest average correlation to the word use in other cities (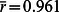), while Minneapolis shows the most unique word use on average, with 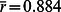.

## Discussion

In this paper we have examined word use in urban areas in the United States, using a simple mathematical method which has been shown to have great flexibility, sensitivity, and robustness. We have used this tool to map areas of high and low happiness and score individual states and cities for average word happiness. In order to understand in greater detail how word usage influences happiness, we used word shift graphs to find the words which produced the greatest difference between the happiness scores of each individual city and the average for the entire US, and socioeconomic census data to attempt to explain the usage of certain words. A significant driver of the happiness score for individual cities was found to be frequency of profanity; we believe that future studies of regional variation in swear word use or ‘geoprofanity’ could help explain geographical differences in happiness. Indeed, swearing has previously been found to be a predictor of large-scale protests and social uprisings in Iran [28].

Happiness within the US was found to correlate strongly with wealth, showing large positive correlation with increasing household income and strong negative correlation with increasing poverty. This is consistent with the first part of the ‘Easterlin paradox’ [29], that within countries at a given time happiness consistently increases with income. The second part of the paradox is that while personal wealth has been observed to consistently increase over time, happiness has tended to decrease in both developed and developing countries [29], [30]. A previous result using our hedonometer method showing a decline in happiness over the 2009–2011 period (see Figure 3 of [11]) is consistent with this finding. The relationship between wealth and happiness is still highly debated; recent works by Stevenson and Wolfers [31] claim to show a direct correlation between gross domestic product and subjective well-being across countries, while Di Tella and MacCulloch [32] in the same year argue that the Easterlin paradox is in fact exacerbated if economic variables other than just income are considered.

We also observed that happiness anticorrelates significantly with obesity. A similar link between obesity and happiness has previously been reported [33], particularly for individuals who report low self control [34]. However, as some authors point out, the presence of chronic illnesses accompanying obesity can confound the link between obesity and psychological well-being [35], and indeed an inverse relationship between weight and depression has been found in some studies [36]. We remark that it should be possible to use techniques such as those described here to mine social network data for real-time surveying. For example, the potential for identifying areas with high obesity based solely on word use is significant.

There are a number of legitimate concerns to be raised about how well the Twitter data set can be said to represent the happiness of the greater population. Roughly 15% of online adults regularly use Twitter, and 18–29 year-olds and minorities tend to be more highly represented on Twitter than in the general population [37]. Furthermore, the fact that we collected only around 10% of all tweets during the calendar year 2011 means that our data set is a non-uniform subsample of statements made by a non-representative portion of the population.

In this work we have only scratched the surface of what is possible using this particular dataset. In particular, we have not examined whether or not these methods have any predictive power–future research could look at how observed changes in the Twitter data set, as measured using the hedonometer algorithm, predict changes in the underlying social and economic characteristics measured using traditional census methods. In particular, we plan to revisit this study when census data for 2012 becomes available to investigate how changes in demographics across urban areas is reflected in happiness as measured by word use.

## Supporting Information

Appendix S1 **Appendices.**
(PDF)
