# Supplementary material for: The Geography of Happiness: Connecting Twitter Sentiment and Expression, Demographics, and Objective Characteristics of Place
Source: PLoS One. 2013 May 29;8(5):e64417. doi: 10.1371/journal.pone.0064417 (PMC3667195; doi:10.1371/journal.pone.0064417)
Supplement: Appendix S1 — Appendices. (PDF) [file pone.0064417.s001.pdf]

Supporting Information - Appendices

A Data set and states

In figure S1 we show the relationship between perimeter and area for the 3592 cities in the MAF/TIGER data database, which follow an approximate power law. The smallest city in both area and perimeter is Richmond, California, while the largest city is New York, whose perimeter extends far north into Connecticut and is agglomerated with Newark, New Jersey in this data set. We find that city area shows an approximate power-law dependence upon perimeter, with an average fractal dimension of $\alpha = 1.294$. Similar results have been reported previously for cities [1, 2], and have even been found to compare well with the fractal dimension of malignant skin lesions [3].

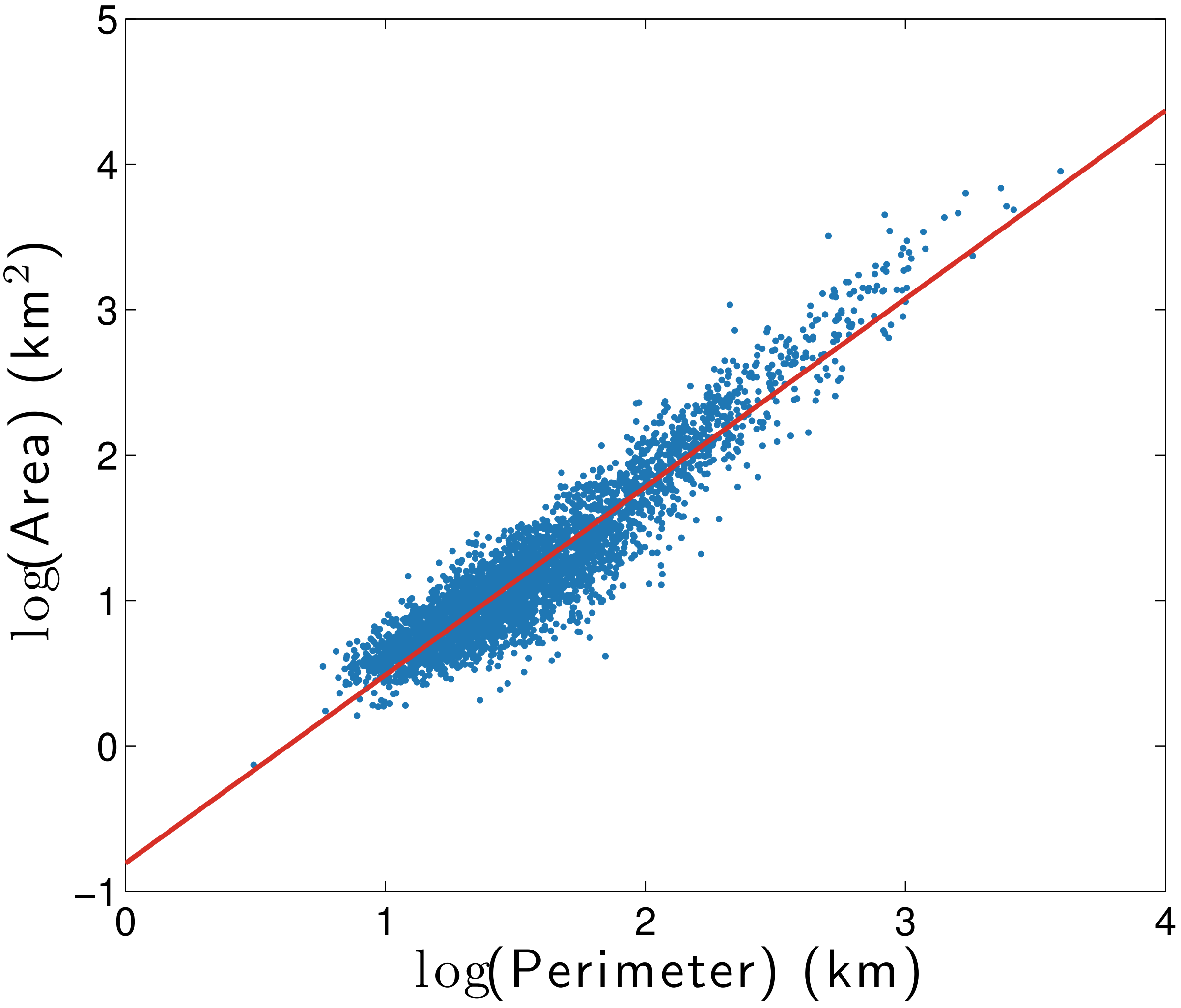

Figure S1. Approximate power law relationship between city area and perimeter for all 3592 cities in the census data set. The fractal dimension is approximately 1.294, the slope of the red trend line.

In preprocessing the Twitter data set we have attempted to remove tweets from users that are clearly automated bots, in particular tweets from weather-recording services which periodically report values of temperature, humidity and the like. Users for whom more than 15% of their tweets contained the words ‘humid’, ‘humidity’, ‘pressure’ or ‘earthquake’ were removed from the dataset. The happiness of individual cities tended to be biased towards the score for each city name (as the name of each city was more likely to be found within that city); to reduce this bias we removed the words ‘atlantic’, ‘grand’, ‘green’, ‘falls’, ‘lake’, ‘new’, ‘santa’, ‘haven’, and ‘battle’ from the cities data set. We also made the decision to remove all variants of the racial pejorative or ‘N-word’ from calculations of h_{avg}. Variants of this word have very low happiness values, averaging $h_{\text{avg}} = 2.92$, and consequently were found to be highly influential in determining the average city happiness. However, when examining individual tweets we found that this word appeared to be being used in conversation as a more colloquial stand in for the word ‘friend’ in the vast majority of cases, and not in fact in any particularly negative sense. As such, we decided that scoring of the word was unfairly biasing our results towards the negative and removed it because of this. Future work will investigate the scoring of phrases instead of words, which will reduce the need for this type of adjustment.

For each city we create the normalized word frequency distribution $\hat{f}(i) = f_i/n$, where n is the total number of tweets collected for that city. The sum $\sum_i^N f_i/n$ therefore represents the average number of LabMT words per tweet, the mean of which is approximately 7.1. In figure S2 we show the average tweet length for the US cities for which we have collected more than 50000 words throughout 2011. Average tweet lengths range from 9 words per tweet for Durham, North Carolina up to almost 12 words per tweet in New York.

Figure S3 shows choropleths for the number of geotagged tweets collected (left) and number of geotagged tweets normalized by state population (right) for the 2011 data set. In both plots the gray scale is logarithmic. In table S1 we show the complete list of happiness scores for all US states. Word shift plots for each state are presented in Appendix B (online) [4].

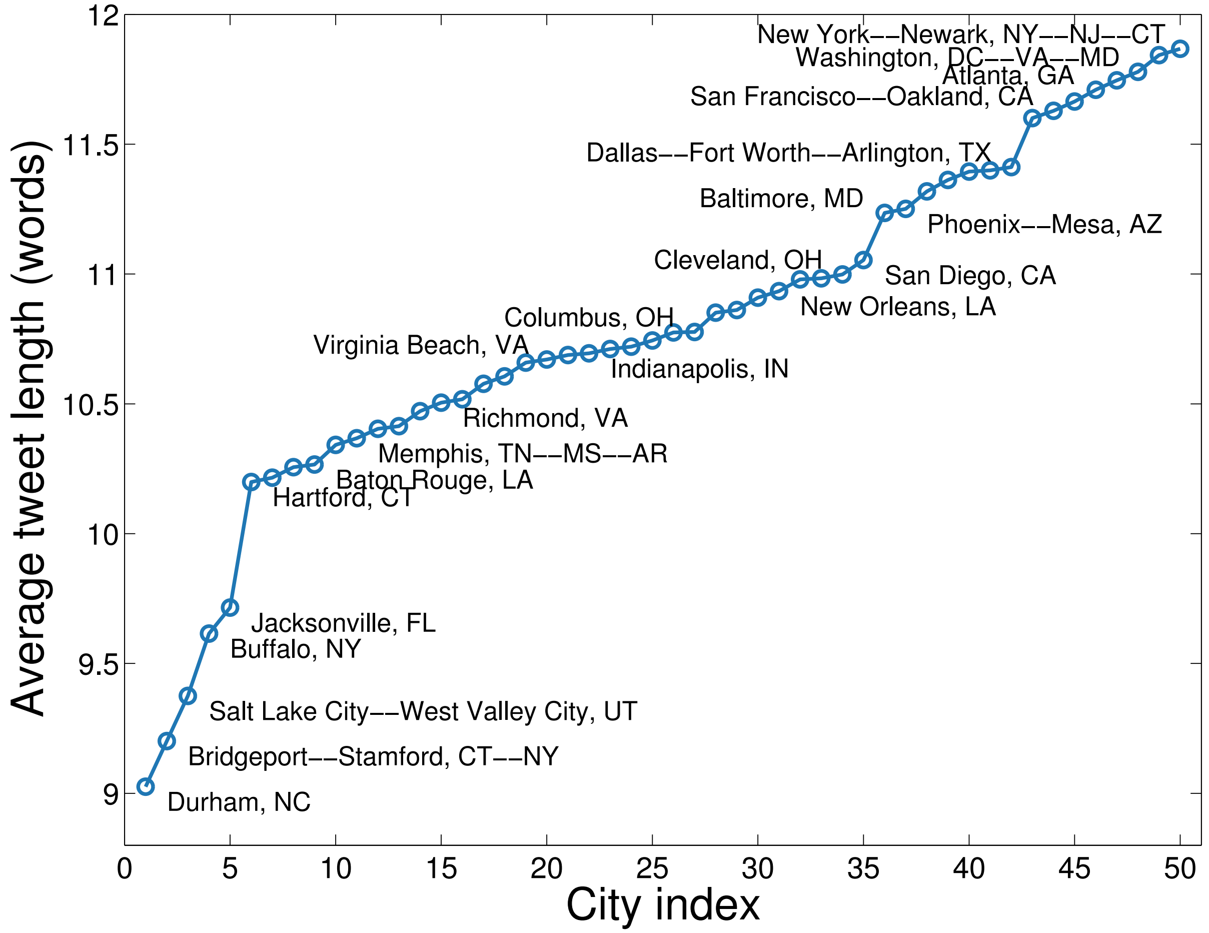

Figure S2. Average message length for US cities with more than 50000 LabMT words collected during 2011.

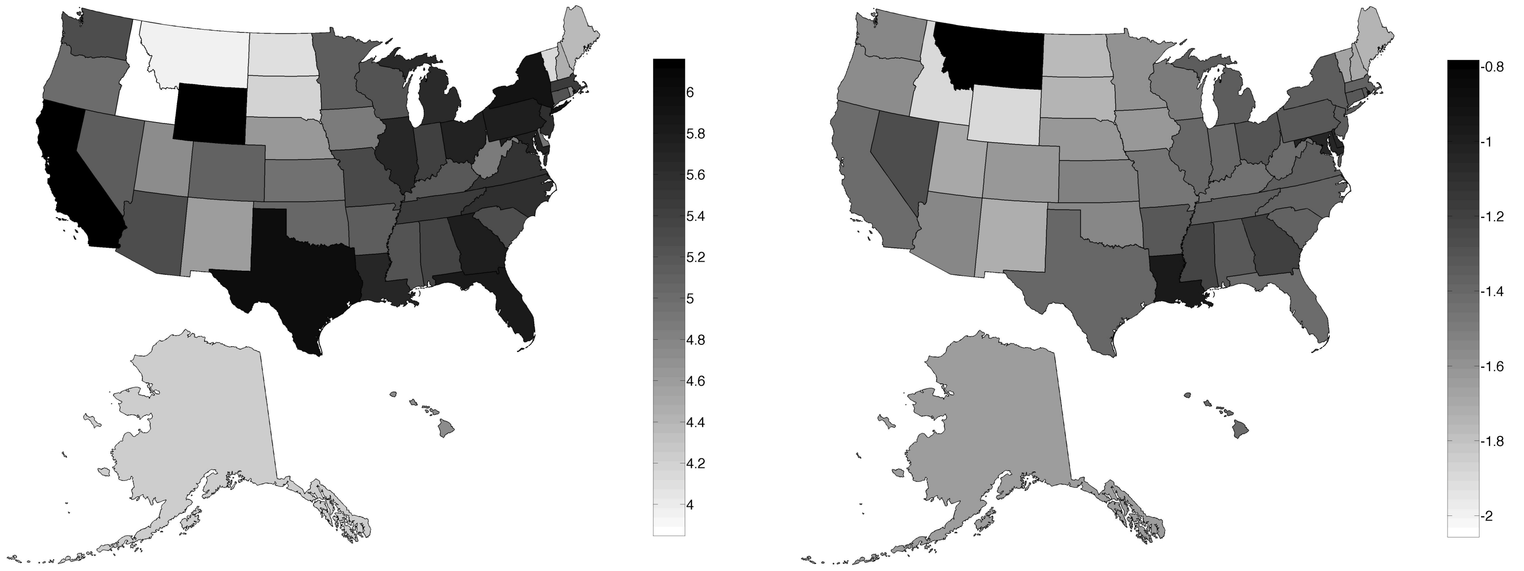

Figure S3. Choropleths showing (the base-10 logarithm of) raw count (left) and number of geotagged tweets collected per capita (right) in each US state during the calendar year 2011.

In tables S2 and S3 we show lists of the top 25 LabMT words with highest positive and negative correlation to obesity, respectively. In table S4 we show the words with lowest correlation to obesity, that is, the words with p-values greater than 0.9. Complete lists for for word correlations with all demographic attributes can be found in Appendix D (online) [4].

B,C,D,E,F Online appendices

The remaining appendices are located online, at http://www.uvm.edu/storylab/share/papers/mitchell2013a/. Appendix B contains word shift graphs for all states, Appendix C contains a comparison between happiness and the Gallup-Healthways well-being measure as well as tweet maps and word shift graphs for all cities, and Appendix D contains complete tables of correlations between demographic attributes and both happiness and word usage. Appendix E contains the complete list of LabMT words ordered by correlation with happiness, and Appendix F is a daily-updating happiness map of the United States.

Rank	State	h_{avg}
1	Hawaii	6.16
2	Maine	6.14
3	Nevada	6.12
4	Utah	6.11
5	Vermont	6.11
6	Colorado	6.10
7	Idaho	6.10
8	New Hampshire	6.09
9	Washington	6.08
10	Wyoming	6.08
11	Minnesota	6.07
12	Arizona	6.07
13	California	6.07
14	Florida	6.06
15	New York	6.06
16	New Mexico	6.05
17	Iowa	6.05
18	Oregon	6.05
19	North Dakota	6.04
20	Nebraska	6.04
21	Wisconsin	6.03
22	Kansas	6.03
23	Alaska	6.02
24	Oklahoma	6.02
25	Massachusetts	6.02
26	Montana	6.01
27	Missouri	6.01
28	Kentucky	6.00
29	New Jersey	5.99
30	West Virginia	5.99
31	Illinois	5.99
32	Rhode Island	5.99
33	Indiana	5.98
34	Texas	5.98
35	South Dakota	5.98
36	Virginia	5.97
37	Tennessee	5.97
38	Connecticut	5.97
39	Pennsylvania	5.97
40	South Carolina	5.96
41	North Carolina	5.96
42	Ohio	5.96
43	Arkansas	5.95
44	District of Columbia	5.94
45	Michigan	5.94
46	Alabama	5.94
47	Georgia	5.94
48	Delaware	5.92
49	Maryland	5.90
50	Mississippi	5.89
51	Louisiana	5.88

Table S1. Happiness scores h_{avg} for each US state, in order from highest to lowest.

Word	ρ	p-value	$h_{\text{avg}}(w_i)$
don't	0.461	2.28×10^{-11}	3.70
give	0.443	1.57×10^{-10}	6.54
lie	0.442	1.68×10^{-10}	2.60
hell	0.438	2.56×10^{-10}	2.22
my	0.438	2.74×10^{-10}	6.16
she	0.433	4.36×10^{-10}	6.18
okay	0.423	1.18×10^{-9}	6.56
like	0.419	1.72×10^{-9}	7.22
girl	0.419	1.76×10^{-9}	7.00
know	0.415	2.54×10^{-9}	6.10
act	0.412	3.48×10^{-9}	6.00
bitch	0.411	4.01×10^{-9}	3.14
me	0.403	8.5×10^{-9}	6.58
all	0.400	1.08×10^{-8}	6.22
nothin	0.399	1.14×10^{-8}	3.64
better	0.398	1.34×10^{-8}	7.00
bored	0.396	1.5×10^{-8}	3.04
bed	0.395	1.72×10^{-8}	7.18
sleep	0.395	1.78×10^{-8}	7.16
wish	0.388	3.25×10^{-8}	6.92
never	0.387	3.43×10^{-8}	3.34
money	0.380	6.41×10^{-8}	7.30
hate	0.378	7.57×10^{-8}	2.34
make	0.376	9.32×10^{-8}	6.00
cant	0.376	9.33×10^{-8}	3.48

Table S2. Top 25 words with strongest positive Spearman correlation ρ to obesity in 2011. Stop words with $4 < h_{\text{avg}} < 6$ have been removed from the list.

Word	ρ	p-value	$h_{\text{avg}}(w_i)$
cafe	-0.509	6.07×10^{-14}	6.78
photo	-0.493	4.87×10^{-13}	6.88
thai	-0.476	3.69×10^{-12}	6.22
fitness	-0.472	5.92×10^{-12}	6.92
park	-0.468	9.59×10^{-12}	7.08
yoga	-0.448	8.82×10^{-11}	7.04
restaurant	-0.448	8.93×10^{-11}	7.06
banana	-0.434	3.77×10^{-10}	6.86
event	-0.433	4.54×10^{-10}	6.12
hotel	-0.429	6.41×10^{-10}	6.16
spa	-0.420	1.54×10^{-9}	6.92
interesting	-0.420	1.62×10^{-9}	7.52
design	-0.409	4.76×10^{-9}	6.32
apple	-0.408	5.22×10^{-9}	7.44
feliz	-0.406	6.47×10^{-9}	6.04
photos	-0.404	7.8×10^{-9}	6.94
wine	-0.400	1.08×10^{-8}	6.42
bike	-0.399	1.22×10^{-8}	6.72
sun	-0.398	1.31×10^{-8}	7.80
delicious	-0.392	2.17×10^{-8}	7.92
flight	-0.391	2.34×10^{-8}	6.06
sunset	-0.391	2.51×10^{-8}	7.16
lounge	-0.389	2.93×10^{-8}	6.50
mortgage	-0.386	3.83×10^{-8}	3.88
dinner	-0.386	3.85×10^{-8}	7.40

Table S3. Top 25 words with strongest negative Spearman correlation ρ to obesity in 2011. Stop words with $4 < h_{\text{avg}} < 6$ have been removed from the list.

Word	ρ	p-value	$h_{\text{avg}}(w_i)$
olive	-0.001	9.94×10^{-1}	6.00
refrigerator	0.001	9.9×10^{-1}	N/A
hashbrowns	0.002	9.83×10^{-1}	N/A
eatting	-0.002	9.76×10^{-1}	N/A
sauteed	0.003	9.72×10^{-1}	N/A
fritos	-0.003	9.69×10^{-1}	N/A
munch	0.003	9.64×10^{-1}	N/A
doughnuts	-0.003	9.62×10^{-1}	N/A
cola	-0.004	9.62×10^{-1}	N/A
okra	-0.004	9.59×10^{-1}	N/A
grapes	0.004	9.51×10^{-1}	N/A
noodles	-0.004	9.51×10^{-1}	N/A
quiznos	0.005	9.49×10^{-1}	N/A
cucumbers	0.005	9.46×10^{-1}	N/A
chow	0.006	9.3×10^{-1}	N/A
walnut	0.007	9.28×10^{-1}	N/A
mulberry	0.007	9.19×10^{-1}	N/A
muesli	0.008	9.17×10^{-1}	N/A
hershey's	0.008	9.17×10^{-1}	N/A
snickers	0.008	9.16×10^{-1}	N/A
krispy	-0.008	9.15×10^{-1}	N/A
nugget	-0.008	9.12×10^{-1}	N/A
smores	0.008	9.1×10^{-1}	N/A
popcorn	0.009	9.07×10^{-1}	6.76

Table S4. The 24 food-related words which show least correlation with obesity, and have p-values greater than 0.9. Words are arranged in decreasing order of p-value.

References

1. White R, Engelen G (1993) Cellular automata and fractal urban form: a cellular modelling approach to the evolution of urban land-use patterns. Environment and Planning A 25: 1175–1199.

2. Shen G (2002) Fractal dimension and fractal growth of urbanized areas. International Journal of Geographical Information Science 16: 419–437.

3. Hern W (2008) Urban malignancy: similarity in the fractal dimensions of urban morphology and malignant neoplasms. International Journal of Anthropology 23: 1–19.

4. Supplementary material for this article is available online at http://www.uvm.edu/storylab/share/papers/mitchell2013a/.
